# Hemothorax after oocyte retrieval in a patient with a history of COVID-19: a case report

**DOI:** 10.5935/1518-0557.20210043

**Published:** 2021

**Authors:** Danilo Rahal, Isadora Ferreira Kozlowski, Vinicius Bonato da Rosa, Alessandro Schuffner

**Affiliations:** 1 Conceber Centro de Medicina Reprodutiva, Curitiba, Paraná, Brazil; 2 Faculdade de Medicina, Pontifícia Universidade Católica, Curitiba, Paraná, Brazil

**Keywords:** hemothorax, oocyte retrieval, coronavirus infection

## Abstract

Spontaneous hemothorax is a rare disorder characterized by pleural fluid hematocrit greater than 50% of the peripheral blood hematocrit without natural or iatrogenic trauma to the lungs or pleural space. Since the first case of COVID-19, more than 85 million cases have been confirmed and most patients have sustained symptoms after more than six months of acute infection. This paper reports the case of a 38-year-old woman without signs of endometriosis and a history of COVID-19 infection who developed spontaneous hemothorax after oocyte retrieval. Three months before undergoing assisted reproductive technology (ART) treatment, the patient had a symptomatic COVID-19 infection with a negative PCR test and a positive IgG test four weeks after the onset of symptoms. Controlled ovarian stimulation and oocyte retrieval were conducted uneventfully. Two hours after oocyte retrieval, the patient developed nausea and mild hypogastric pain. Ten hours after the procedure, the patient went to the emergency department with abdominal pain. Chest computed tomography scans revealed moderate right pleural effusion and laminar left pleural effusion. Since the patient had respiratory symptoms, the choice was made to drain the pleural fluid. Fluid analysis confirmed the patient had right hemothorax (400 mL). After drainage, the patient's clinical and imaging signs improved gradually without complications. The patient was asymptomatic one week after the procedure.

## INTRODUCTION

Infertility affects about 15% of couples of reproductive age ^(Alegretti *et al*., 2013)^. Spontaneous hemothorax is a rare disorder characterized by pleural fluid hematocrit greater than 50% of the peripheral blood hematocrit without natural or iatrogenic trauma to the lungs or pleural space ^(Morgan *et al*., 2015)^. Spontaneous hemothorax is a rare disorder, with an incidence between 2.0-7.3%, affecting mainly men aged 15-39 years ^(Long & Grimaldo, 2021)^.

In December 2019, the first case of COVID-19 was identified in Wuhan, China, and on March 11, 2020, the disease was declared a pandemic by the World Health Organization (WHO) ^(Long & Grimaldo, 2021)^. Since the discovery of COVID-19, more than 85 million cases have been confirmed with more than 1.8 million deaths from the disease ^(WHO, 2021)^. A recent study showed that 76% of the evaluated patients sustained symptoms after more than six months of acute infection; women are preferentially affected; and 22% to 56% of the patients had changes in lung diffusion six months after the onset of symptoms ^(Huang *et al*., 2021)^.

The presence of spontaneous hemothorax in patients with COVID-19 is a very rare presentation ^(Halvorson *et al*., 2012; Sopa *et al*., 2017)^, and the cases reported occurred during the acute phase of the disease ^(Long & Grimaldo, 2021)^. The association of spontaneous hemothorax with in vitro fertilization (IVF) procedures is rare and has been associated with thoracic endometriosis.

This paper reports the case of a patient without signs of endometriosis and a history of COVID-19 infection who developed spontaneous hemothorax after oocyte retrieval.

## CASE DESCRIPTION

The patient is a 38-year-old nulligravida with a BMI of 21.72 and without comorbidities who had been trying to get pregnant for three years. She had normal hysterosalpingography findings, ultrasound examination showing no signs of deep endometriosis, anti-Müllerian hormone (AMH) levels 11 months before treatment of 2.0 ng/mL, and normal results in other related tests. Her partner had secondary infertility, a spermogram showing increased sperm viscosity, mild left varicocele, and minor bilateral hydrocele.

One month prior to IVF, the patient's complete blood count showed the following results: Hb 12.6 g/dL; GV 37.7%; 7,260 leukocytes/mm^3^; and 274,000 platelets/mm^3^.

The couple chose to undergo IVF with preimplantation genetic testing for aneuploidy (PGTA) due to the age of the female partner.

The patient had COVID-19 three months prior to IVF, with symptoms of myalgia, fever, and retro-orbital headache; her PCR test was negative. She was treated for symptoms only. However, since suspicions of infection by SARS-CoV-2 remained, she had an IgG test four weeks later, which confirmed she had COVID-19. The patient progressed well after the acute phase of the disease.

Ovulation induction was initiated on the third day of the menstrual cycle with follitropin alfa 225 IU and menotropin 75 IU daily. Serial ultrasound was performed to monitor ovarian follicles. On the seventh day of the cycle, when the largest follicle reached 14mm in average diameter, the patient was started on GnRH antagonist cetrorelix 0.25mg a day. On the tenth day of the cycle, a double trigger was performed, with 250mcg of choriogonadotropin alfa and 0.2mg of triptorelin acetate.

On the day of trigger, there were 13 follicles above 12mm in average diameter. Oocyte retrieval was performed 35 hours after the trigger. Thirteen mature oocytes, five immature oocytes, and one degenerate oocyte were aspirated. ICSI was performed on all mature oocytes. Twelve fertilized and three evolved into a blastocyst to be biopsied and frozen by vitrification. Of the three biopsied embryos, one had X monosomy, one had mosaic monosomy 4, and the other was normal.

For the oocyte retrieval procedure, anesthesia was performed with propofol and fentanyl and to manage symptoms (ketoprofen, dexamethasone, dipyrone and ondansetron). The entire standardized technique for follicular aspiration was performed using a single lumen needle with an internal diameter of 17G and 33cm in length, guided by transvaginal ultrasound. The procedure was carried out without complications.

Two hours after the procedure, the patient developed nausea and mild hypogastric pain, which worsened in an orthostatic position. One hour later the patient improved from the symptoms, became asymptomatic, and was discharged.

**Figure 1:**
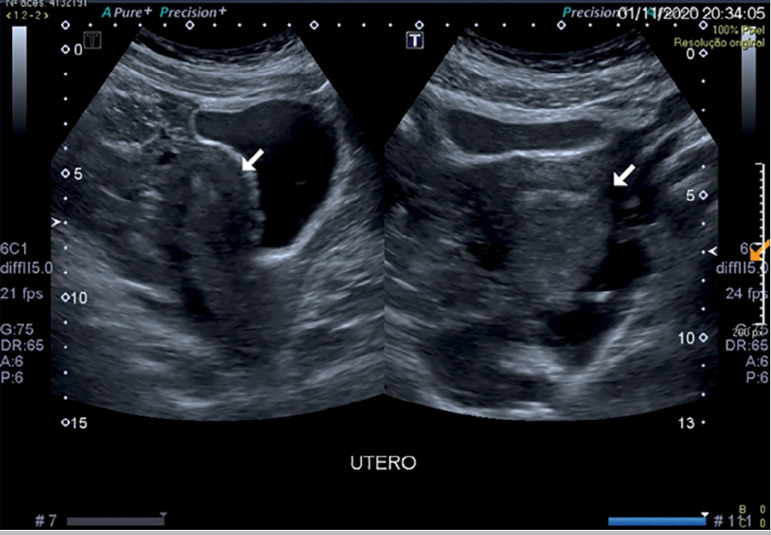
Pelvic ultrasound 12 hours after oocyte retrieval.

**Figure 2:**
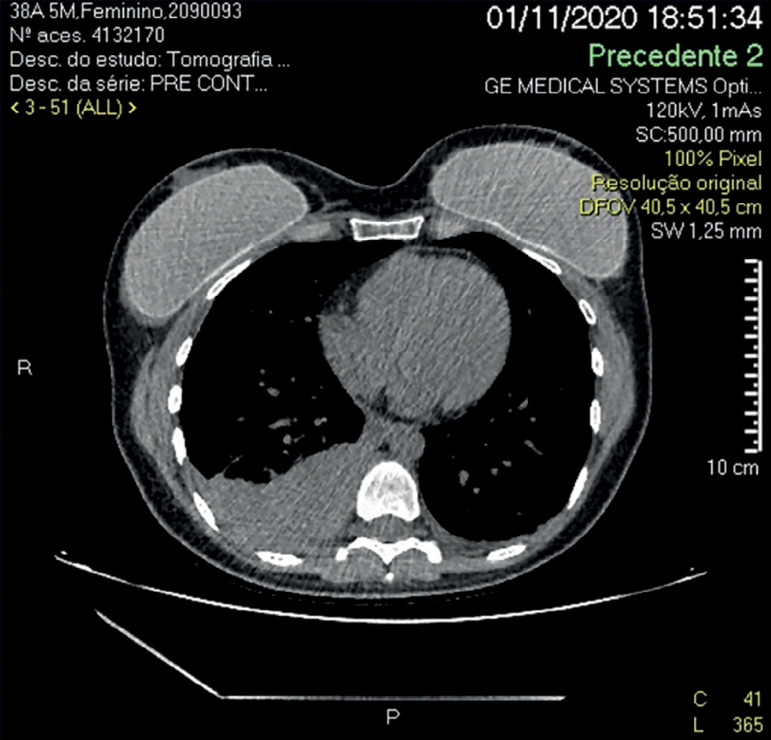
Computed tomography scan showing hemothorax, 10 hours after oocyte retrieval.

Ten hours after the procedure, the patient was admitted to the emergency department with complaints of abdominal pain. A complete blood count was performed, showing Hb 10.2g/dL, GV 28.7%, with 11,060 leukocytes/mm^3^ and 229,000 platelets/mm^3^. Transvaginal ultrasound (Figure 1) and total abdomen tomography (Figure 2) were performed, showing enlarged ovaries, consistent with a recently performed controlled ovarian stimulation, and a small amount of free intra-abdominal fluid, with no signs of active bleeding. Chest computed tomography scans revealed moderate right pleural and laminar left pleural effusion, and small, elongated non-calcified single bilateral pulmonary nodules of up to 4 mm with benign characteristics, probably lymph nodes.

On the following day, since the patient had respiratory symptoms, the choice was made to drain the pleural fluid (performed by a thoracic surgeon). Fluid analysis confirmed the patient had right hemothorax (400mL). Fluid oncotic culture and cytology came back negative. After drainage, the patient's clinical and imaging signs improved gradually without complications. The patient was asymptomatic one week after the procedure.

## DISCUSSION

This report describes the case of a patient with infertility and a history of COVID-19 submitted to IVF who developed spontaneous hemothorax, a development until now not described in the literature.

In cases of ovarian hyperstimulation syndrome (OHSS), patients may have pleural effusion due to exudate, associated with ascites. The early form occurs 3 to 7 days after the administration of human chorionic gonadotropins (hCG) ^(Irani *et al*., 2018)^, which does not fit the case described.

There is a report of hemothorax in a patient submitted to IVF, which occurred in association with severe endometriosis with involvement of the diaphragm ^(Sopa *et al*., 2017)^.

Spontaneous hemothorax is rare in patients with acute COVID-19, although it may occur in some cases ^(Long & Grimaldo, 2021)^. We were unable to find other publications describing cases of spontaneous hemothorax after late COVID-19.

Could COVID-19 leave pulmonary and/or pleural scars that might cause spontaneous hemothorax after surgical stress or exposure to hormones in ovarian stimulation?
